# Effects of age and blood pressure on the retinal arterial wall, analyzed using adaptive optics scanning laser ophthalmoscopy

**DOI:** 10.1038/srep12283

**Published:** 2015-07-20

**Authors:** Shigeta Arichika, Akihito Uji, Sotaro Ooto, Yuki Muraoka, Nagahisa Yoshimura

**Affiliations:** 1 The Department of Ophthalmology and Visual Sciences, Kyoto University Graduate School of Medicine, Kyoto 606-8507, Japan

## Abstract

The wall-to-lumen ratio (WLR) of the vasculature is a promising early marker of retinal microvascular changes. Recently, adaptive optics scanning laser ophthalmoscopy (AOSLO) enabled direct and noninvasive visualization of the arterial wall. Using AOSLO, we analyzed the correlation between age and WLR in 51 normal subjects. In addition, correlations between blood pressure and WLR were analyzed in 73 subjects (51 normal subjects and 22 hypertensive patients). WLR showed a strong correlation with age (r = 0.68, *P* < 0.0001), while outer diameter and inner diameter did not show significant correlation with age in the normal group (r = 0.13, *P* = 0.36 and r = −0.12, *P* = 0.41, respectively). In the normal and hypertensive groups, WLR showed a strong correlation with systolic and diastolic blood pressure (r = 0.60, *P* < 0.0001 and r = 0.65, *P* < 0.0001, respectively). In conclusion, AOSLO provided noninvasive and reproducible arterial measurements. WLR is an early marker of morphological changes in the retinal arteries due to age and blood pressure.

Retinal vessels serve as informative models of microvasculature changes in systemic diseases. For example, the Keith-Wagener^1^ and Scheie^2^ classification systems are the gold standards for directly evaluating and categorizing retinal vascular changes associated with hypertension and arteriosclerosis. However, these classifications are based solely on qualitative parameters, such as vascular color tone, attenuation, tortuosity, arteriovenous crossings, caliber, and optic disc. The imaging software Retinal Analysis-Interactive Vessel Analysis (IVAN) (University of Wisconsin, Madison, Wisconsin, USA) conducts semi-automated vessel measurements from fundus photographs and has been established as the gold standard for quantitative vascular measurements^3^,^4^,^5^. However, it cannot evaluate the retinal vascular wall.

Recently, noninvasive approaches for evaluating the retinal microvasculature have been reported, including scanning laser Doppler flowmetry (SLDF)^6^,^7^,^8^,^9^,^10^ and optical coherence tomography (OCT)^11^. SLDF allows for evaluation of arteriolar morphology by measuring the outer diameter (OD), lumen diameter, wall-to-lumen ratio (WLR), and wall cross-sectional area with automatic perfusion imaging analyses. Furthermore, Rizzoni *et al.* reported that retinal arteriole WLR measured by SLDF was closely correlated with the media-to-lumen ratio of subcutaneous small arteries evaluated by invasive surgical intervention^7^. Harazny *et al.* reported that the WLR in patients with a past cerebrovascular event was significantly increased compared to that in treated hypertensive patients and normotensive subjects^8^.

Adaptive optics (AO) technology, first applied to astronomy, is another promising approach for direct and noninvasive retinal arterial wall visualization^12^,^13^,^14^. AO scanning laser ophthalmoscopy (AOSLO) provides high-resolution imaging of the retinal photoreceptor^15^,^16^, nerve fiber layer^17^, and blood flow^18^,^19^,^20^,^21^. In 2012, Chui *et al.* first succeeded in visualizing the retinal wall with AOSLO^12^. Subsequently, Koch *et al.* reported vascular morphometric changes measured with an AO retinal camera^14^.

In this study, we directly and noninvasively analyzed the vascular wall in hypertensive patients using AOSLO.

## Results

**Figure 1:**
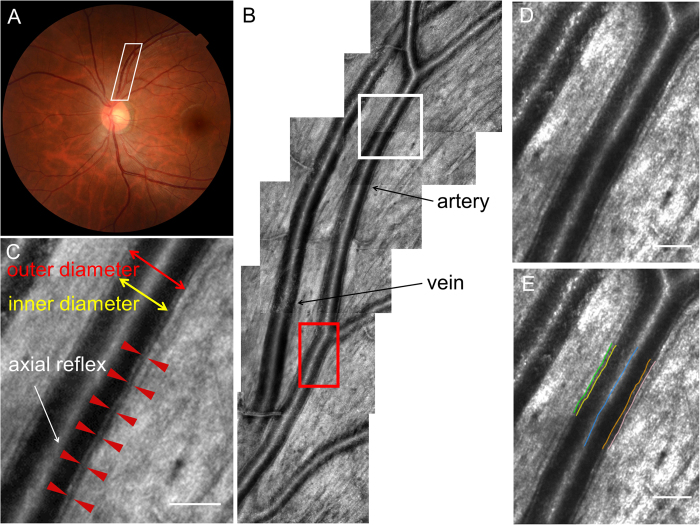
The visualization of the vascular wall using adaptive optics scanning laser ophthalmoscopy. (**a**) The fundus photograph of normal left eye. (**b**) The adaptive optics images in the area outlined in white on panel (**a**). The arterial wall was successfully visualized along the artery. However, the venous wall could not be visualized. (**c**) Magnified images outlined in white on panel (**b**). The interval between the tips of the 2 red arrowheads represents the arterial wall. Scale bar, 100 μm. (**d**) Magnified images outlined in red on panel (**b**). Scale bar, 100 μm. (**e**) The same magnified image as in panel (**d**) with the result from semi-automatic segmentation of vessel border lines. Retinal arterial wall borders were detected along the vessel’s running direction. After control points for the retinal arterial axes were set manually (blue line), vascular wall border detection was processed automatically Green and pink line represent outer wall borders. Yellow and orange line represent inner wall borders. Scale bar, 100 μm.

**Figure 2:**
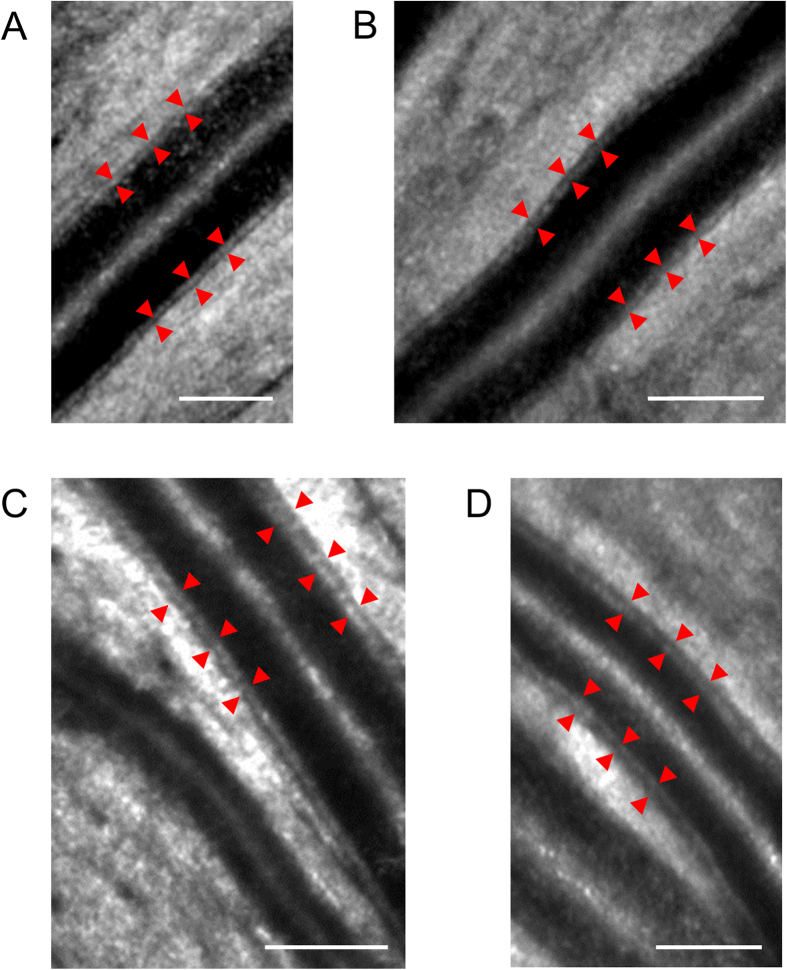
Adaptive optics scanning laser ophthalmoscopy images for wall visualization. (**a**) A 24-year-old woman with a blood pressure of 89/55 mmHg and normal wall thickness. The wall-to-lumen ratio (WLR) was 0.176, and the wall thickness (WT) was 20.2 μm. Red arrows indicate the arterial wall. Scale bar, 100 μm. (**b**) A 43-year-old man with a blood pressure of 118/73 mmHg and normal wall thickness. The WLR was 0.204, and the WT was 20.9 μm. Scale bar, 100 μm. (**c**) A 61-year-old man with a blood pressure of 154/92 mmHg and a thickening wall. The WLR was 0.321, and the WT was 31.7 μm. Scale bar, 100 μm. (**d**) A 57-year-old man with a blood pressure of 140/86 mmHg and a thickening wall. The WLR was 0.332, and the WT was 32.7 μm. Scale bar, 100 μm.

AOSLO provided clear wall visualization of retinal arterial vessels but not venous vessels (Figs 1 and 2). Mean vascular measurements (OD, inner diameter (ID), wall thickness (WT), and WLR) were determined by segment to minimize the cardiac cycle’s influence. Detailed characteristics of both groups are shown in Table 1.

### Reproducibility of vascular measurements by AOSLO

Inter-evaluator intraclass correlation coefficients (ICCs) showed good agreement; the ICCs for OD, ID, WT, and WLR were 0.980, 0.970, 0.889, and 0.882, respectively. Inter-visit ICCs also showed good agreement; the ICCs for OD, ID, WT, and WLR were 0.961, 0.952, 0.977, and 0.960, respectively.

### Vascular caliber differences between normal and hypertensive patients

**Figure 3:**
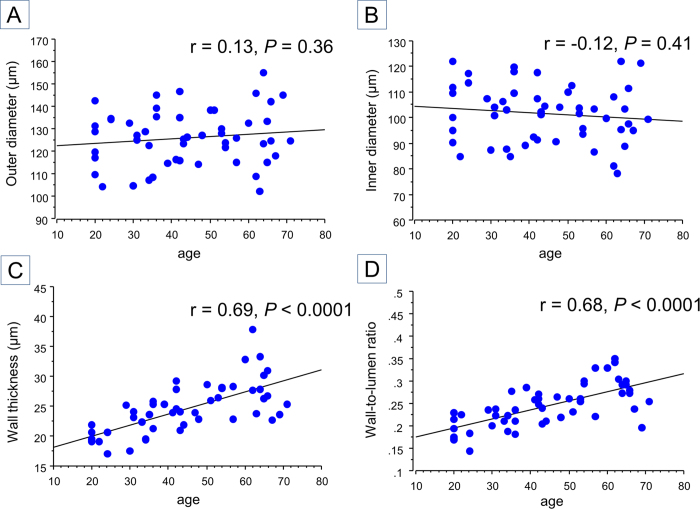
The relationship between age and vascular measurements in normal subjects. (**a**,**b**) Outer diameter and inner diameter did not show significant correlation with age (r = 0.13, *P* = 0.36 and r = −0.12, *P* = 0.41, respectively). **(c**,**d**) However, wall thickness and wall-to-lumen ratio showed significant correlation with age (r = 0.69, *P* < 0.0001 and r = 0.68, *P* < 0.0001, respectively).

In the normal group, the average OD, ID, WT, and WLR were 126.2 ± 12.3 μm, 101.6 ± 11.2 μm, 24.5 ± 4.3 μm, and 0.244 ± 0.047, respectively. OD and ID were not significantly correlated with age (r = 0.13, *P* = 0.36, and r = −0.12, *P* = 0.41, respectively) (Fig. 3), but WT and WLR were (r = 0.69, *P* < 0.0001, and r = 0.68, *P *< 0.0001, respectively). In the hypertensive group, the mean OD, ID, WT, and WLR were 122.3 ± 18.7 μm, 93.1 ± 15.6 μm, 29.2 ± 5.6 μm, and 0.320 ± 0.068, respectively. To adjust for age between groups, and because of the limited prevalence of hypertension in younger patients, we compared subjects aged >50 years between groups (Table 2). There was a significant difference in WLR (*P* = 0.04) but not in OD, ID, and WT (*P* = 0.21, 0.09, and 0.50, respectively).

### Correlation between vascular caliber and blood pressure parameters

Among all 73 subjects, OD showed no significant correlation with systolic blood pressure (SBP), diastolic blood pressure (DBP), age, body mass index (BMI), pulse pressure (PP), and heart rate (HR). ID was significantly correlated with SBP and DBP, but not with age, BMI, PP, and HR. WT and WLR were significantly correlated with SBP, DBP, age, BMI, and PP, but not with HR (Table 3). We then used a multivariate regression analysis with a backward stepwise method to analyze predictors of WLR with regard to SBP, DBP, age, BMI, and HR. DBP and age were independent variables for WLR (Table 4).

### Correlation between AOSLO-based and fundus photograph-based vascular measurements

The mean arterial diameter calculated by the IVAN system was 96.8 ± 9.9 μm in the normal group and 93.1 ± 14.4 μm in the hypertensive group. Four subjects were excluded because of difficulties with IVAN measurements. Calibers did not significantly correlate with age in either group (normal group, r = 0.14, *P* = 0.36; hypertensive group, r = 0.17, *P* = 0.45). Among the 69 subjects included in this analysis, IVAN-measured diameter was not significantly correlated with SBP, DBP, BMI, PP, or HR. We evaluated vascular caliber (OD and ID) measured by IVAN and AOSLO in both the normal and hypertensive groups using the same artery and found a strong correlation (r = 0.65, *P *< 0.0001 for OD, and r = 0.63, *P *< 0.0001 for ID).

## Discussion

In this study, AOSLO provided noninvasive visualization and measurement of retinal arterial caliber with high reproducibility and validity. WLR was highly positively correlated with both age and blood pressure.

Pathophysiologically, small arterial remodeling, which is the first sign of hypertension, consists of inward eutrophic remodeling and outward hypertrophic remodeling^22^,^23^. Eutrophic remodeling reflects blood pressure stress and myogenic vasoconstriction; therefore, only the WLR increases. In contrast, hypertrophic remodeling reflects the secondary stimulus of arterial hypertension; therefore, both WLR and wall cross-sectional area increase. Thus, WLR is a promising early marker of retinal microvascular changes, reflecting retinal endothelial dysfunction in systemic diseases^24^. In this study, AOSLO showed a strong correlation between WLR and SBP or DBP. Meanwhile, ID showed modest correlation with SBP and DBP, and OD showed no significant correlation with either. Moreover, multivariate regression analysis revealed DBP and age as independent variables for WLR. Ritt *et al.* reported that retinal arteriole WLR by SLDF was significantly positively correlated with SBP and DBP^25^. Koch *et al.* reported that mean blood pressure was negatively correlated with ID using an AO camera. Moreover, mean blood pressure was positively correlated with parietal and WLR, with stronger statistical significance than that for ID^14^. These data support the potential use of WLR as an early marker of retinal microvascular changes.

While arteriolar diameter decreased with age in a photograph study^26^, ID and OD by AOSLO were not significantly correlated with age, and WT and WLR correlated well with age in the normal group. In agreement with our AOSLO data, by SLDF, Harazny *et al.* showed positive correlation between age and WLR in normotensive individuals^8^, and Michelson *et al.* demonstrated the influence of age on the arterial wall in normal subjects^27^. Using OCT, Muraoka *et al.* demonstrated that age significantly correlated with increased OD and increased WT, but not with ID^11^. Taken together, WT and WLR show significant positive correlation with age and are promising indicators of vascular changes.

To date, other modalities have also been used for arterial measurements. Using SLDF, OD, ID, unilateral WT, and WLR have been reported as 93.6 μm, 74.4 μm, 9.6 μm, and 0.264, respectively^7^; 109 μm, 85.3 μm, 12.0 μm, and 0.28, respectively^25^; and 110 μm, 82.3 μm, 14.0 μm, (WLR was not listed), respectively^27^. Using OCT, OD, ID, and unilateral WT were previously reported as 122.7 μm, 87.3 μm, and 17.7 μm, respectively^11^. Using an AO camera, ID, WT, and WLR were 83.5 μm, 23.5 μm, and 0.285, respectively^14^. Despite the slight variation in vascular diameters, WLR values were similar using SLDF, AO camera, and AOSLO. Here, only WLR was significantly different between groups, while OD, ID, and WT were not (Table 2). Ritt *et al.* reported that both WT and WLR of retinal arterioles in the hypertensive group, but not OD and ID, were statistically higher than those in the normal group^25^. In contrast, Rizzoni *et al.* reported that OD, ID, and WLR, but not WT, in the hypertensive group were statistically higher than those in the normal group^7^. Using OCT, OD, ID, and unilateral WT were 125.2 μm, 88.5 μm, and 18.3 μm^11^, respectively, and only WT was significantly different compared to the normal group. Using an AO camera, ID, bilateral WT, and WLR were 74.0 μm, 25.5 μm, and 0.36^14^. ID and WLR, but not WT, showed significant differences compared to the normal group. It is generally difficult to compare vascular calibers measured by different modalities for the possible reasons described below. First, the measuring points and measured arteries were slightly different among studies. Here, the largest temporal arteries were selected in the zone B, 0.5–1.0 disc diameters away from the optic disc margin^28^. The SLDF measuring point was 1 disc diameter temporally superior from the optic disc margin; the OCT measuring point was 1.73 mm from the center of the optic disc on the four largest arteries; and the AO camera measuring point was 1 disc diameter temporally superior from the optic disc margin. Second, the targets for vascular measurements were different. IVAN measurements were thought to reflect lumen diameter. However, the calibers on the color photograph reflect the moving blood column, which is surrounded by the transparent plasma edge stream. Therefore, calibers on the photograph underestimate the true internal diameter^23^. The same principle may be applied to SLDF. Laser Doppler imaging, thought to reflect flow diameter as lumen diameter, consists of blood corpuscles surrounded by the plasma edge stream^27^. In addition, the scanning directions for imaging were different among the modalities. OCT provides vertical vessel information, while photographs, SLDF, AO camera, and AOSLO provide horizontal information. Furthermore, there are the other possible factors for these differences in retinal arterial vessel measurements. The mean subject ages varied in each study. Mean age in the SLDF study ranged from 36.7 to 59.3 years^7^,^25^,^27^,^29^. In the OCT study, the mean age was 62.1 years^11^, and that in the AO camera study was 44.9 years^14^. The mean age here was 44.3 years. Furthermore, data reported using other modalities were calculated without correction for axial length; therefore, the actual distance per pixel differed from eye to eye.

There were certain limitations to this study. For example, there were few subjects aged >70 years, because AOSLO image quality is influenced by cataracts. In the future, additional studies using larger sample sizes will be necessary to confirm our results. The other limitation is the lack of comparison with other retinal arterial wall measurements, such as SLDF or the micromyographic approach, because of their limited availability. In the near future, we will compare retinal wall measurements noninvasively and directly between OCT and AOSLO.

AOSLO provided reproducible noninvasive arterial wall measurements. WLR was significantly and positively correlated with age and blood pressure, suggesting that WLR has the potential to be an early parameter of retinal vascular microchanges. Furthermore, AOSLO arterial measurements showed a high correlation with vascular measurements by fundus photograph. This new approach will enable evaluation of early morphological changes of retinal microvasculature due to age and blood pressure.

## Methods

This study was approved by the Institutional Review Board and Ethics Committee at Kyoto University Graduate School of Medicine and adhered to the tenets of the Declaration of Helsinki. After the study design and the risks and benefits of participation were thoroughly explained, written informed consent was obtained from each participant.

### Participants

AOSLO movies were acquired for 51 normal subjects (26 men, 25 women) and 22 hypertensive patients (16 men, 6 women). Either eye, dilated before AOSLO with one drop of tropicamide (0.5%) and phenylephrine hydrochloride (0.5%), was selected for this analysis. The exclusion criteria were as follows: best-corrected visual acuity worse than 20/25; high myopia (more severe than -6 diopters and longer than 26.0 mm in axial length); intraocular pressure >21 mmHg; pregnancy; diabetes mellitus; renal failure; any form of secondary arterial hypertension; history of cerebral infarction; history of myocardial infarction; ocular diseases except hypertensive retinopathy; and systemic diseases except for hypertension. Subjects were examined for approximately 15 min in total per eye while in a seated position.

### AOSLO imaging

**Figure 4:**
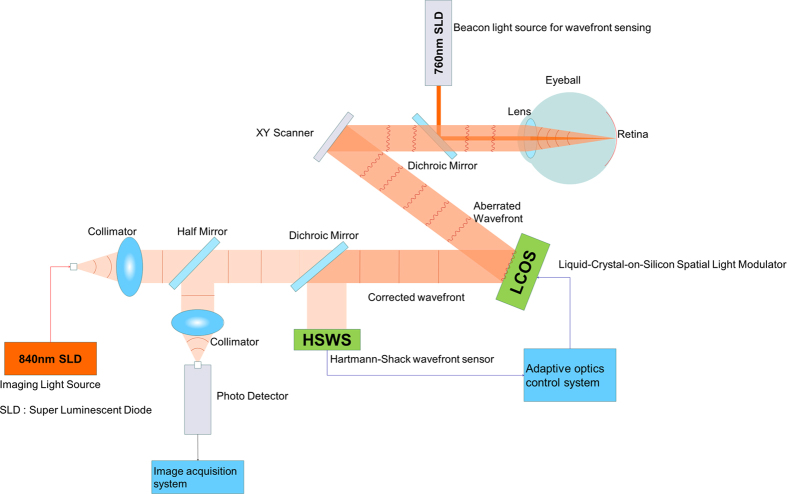
Schematic of adaptive optics scanning laser ophthalmoscopy. A spatial light modulator, based on the Liquid-Crystal-on-Silicon, and a wavefront sensor, based on the Shack-Hartmann sensor, was used to compensate for wavefront errors. The high-resolution imaging system was a confocal scanning laser ophthalmoscopy with light emitted from an 840-nm superluminescent diode.

The AOSLO system (Canon Inc., Tokyo, Japan) is composed of the AO system, a high-resolution confocal SLO imaging system, and a wide-field imaging subsystem (Fig. 4). The resolution of our system is 2 μm/pixel. Video was recorded for 10 s per scan area at a rate of 32 frames/s. AOSLO imaging was performed with the optical focus on the layer for which the wall could be appropriately visualized. As Bennett *et al.* reported previously^30^, each subject’s axial length, obtained with an optical biometer (IOL Master; Carl Zeiss Meditec, Dublin, CA), was used to convert the degree to the actual distance to the retina using AOSLO Retinal Image Analyzer software (ARIA; Canon Inc., Tokyo, Japan) dedicated to our prototype AOSLO^31^. A pulse oximeter (Oxypal Neo; NIHON KOHDEN, Japan) was attached to subjects’ earlobes for synchronizing cardiac pulsation and AOSLO video frames. The sphygmogram was digitized and recorded during the imaging session. The ARIA software detected extreme values from the sphygmogram and determined the relative cardiac cycle for each frame of the captured AOSLO video^31^.

### Blood pressure parameters

Hypertension was defined as mean SBP ≥140 mmHg, mean DBP ≥90 mmHg, taking antihypertensive medication at the time of examination, and/or a physician’s diagnosis^32^. SBP and DBP were measured thrice to provide means for this analysis. Blood pressure was measured on the examination day in subjects who had been seated for at least 5 minutes. PP was defined as SBP - DBP.

### Retinal arterial vascular caliber measurements

#### AOSLO

**Figure 5:**
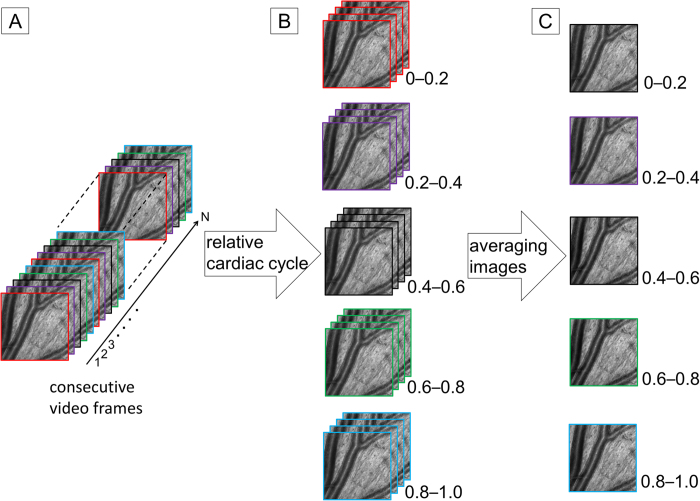
Calculation of the mean vascular measurements correcting for cardiac pulsation. In order to minimize the influence of cardiac pulsation on vascular measurements, the cardiac cycle was synchronized to the adaptive optics (AO) videos using pulsation data obtained through a pulse oximeter attached to the subjects’ earlobes. (**a**) The cardiac cycle was divided into 5 segments (0–0.2, 0.2–0.4, 0.4–0.6, 0.6–0.8, and 0.8–1.0), and each video frame was assigned to the corresponding segment. (**b**) The images on the same relative cardiac cycle were extracted from the AO videos. (**c**) The images of each corresponding segment were averaged, and the vascular caliber measurements were obtained for every 5 segments. Finally, the vascular measurements of 5 segments were averaged and used for analyses.

Vascular caliber measurement was performed in zone B^28^, 0.5–1.0 disc diameters away from the optic disc margin using custom software known as ARIA, which was developed by Canon Inc., Tokyo, Japan. ARIA semi-automatically segments the retinal arterial wall borders in the AOSLO video. Our segmentation method consisted of preprocessing, rough central axis setting, precise central axis and vascular wall border detection, and measurement of the thickness of the vascular wall. Preprocessing consisted of the following steps. Pixel intensity values in the AOSLO video were transformed logarithmically to increase contrast in a vascular wall and decrease it in a nerve fiber layer. The AOSLO video was then stabilized using the inverse scan-line warping method. In order to obtain a high-contrast vascular wall image, frames of the stabilized AOSLO video corresponding to the specific phase range of the pulse wave were selected, and these were then smoothed using a 3-D median filter. We used a filter size of 3 × 3 × (selected frame numbers). In the next step, we manually set seed points at the center of the retinal artery along its running direction (Supplementary Figure S1A). At each seed point, the line segment that was perpendicular to the running direction of the seed points was set, and its pixel intensity profile was calculated. In the precise central axis and vascular wall border detection step, we applied a sliding linear regression filter (SLRF)^33^,^34^ to the pixel intensity profile along the line segment (Supplementary Figure S1B). The SLRF method is based on the fitting of a line by linear regression that relates the pixel intensity value to the distance along the profile within a sliding window (window size: W). The precise position of the vascular central axis was identified around the seed point as a zero-cross point in the pixel intensity profile filtered by SLRF. To determine the vascular edge position, we specified the minimum point in the left side and the maximum point in the right side from the zero-cross point corresponding to the central axis. In order to determine the vascular wall border position more robustly and precisely, we applied SLRF twice to the pixel intensity profile along the line segment that was perpendicular to the central axis. Specifically, SLRF with a larger window size (W = 10 pixels) was applied first, followed by SLRF with a smaller window size (W = 4 pixels). The initial, larger window size (W = 10 pixels) was applied to establish the approximate position of the vascular wall region. In the pixel intensity profile filtered by SLRF, we could establish the approximate position of the vascular wall region by detecting the minimum point in the left side and the maximum point in the right side from the zero-cross point corresponding to the central axis (the minimum and maximum points are shown in Supplementary Figure S2C). Subsequently, we deleted outliers in the first candidate points if their Euclid distance from the central axis was less than the lower threshold (Tl)1 percentile or more than the higher threshold (Th)1percentile (Supplementary Figure S1C). In this study, Tl1 and Th1 were set as 10 and 90, respectively. The remaining first candidate points were interpolated using the natural spline interpolation method (Supplementary Figure S1D). The interpolated candidate points indicated the approximate position of the vascular wall region and were used to calculate the position of the vascular wall border by applying it to the second step (W = 4 pixels) (Supplementary Figure S1E). In the pixel intensity profile filtered by SLRF, we select the 2 nearest extremal points to the candidate point as vascular wall border candidate points (inner and outer borders are shown in Supplementary Figure S2D). Subsequently, we deleted outliers in the vascular wall border candidate points if their pixel intensity values were less than the Tl2 percentile or more than the Th2 percentile, and then deleted the remaining vascular wall border candidate points if their pixel intensity values were less than the Tl3 percentile or more than the Th3 percentile (Supplementary Figure S1F). In this study, Tl2 and Tl3 were set as 10, and Th2 and Th3 were set as 90. The remaining vascular wall border candidate points were sampled at intervals, and then interpolated using the natural spline interpolation method (Supplementary Figure S1G). Finally, we used the interpolated candidate points to define the vascular wall border in calculating the retinal arterial wall thickness (Supplementary Figure S1H). Continuous measurements were performed automatically at 6 μm intervals along the segmented border lines. OD was defined as the distance between the 2 outer wall borders, and ID was defined as the distance between the 2 inner wall borders. WLR was calculated as WT/ID^7^,^8^. In order to minimize the influence of cardiac pulsation on vascular measurements, an averaged image was generated using stabilized frames with a specific range of relative cardiac cycles (Fig. 5). This enabled division of the AOSLO images into 5 segments according to cardiac pulsation, and mean vascular measurements were obtained for every 5 segments. Finally, vascular measurements of 5 segments were averaged and used for analyses.

#### Fundus photographs

Forty-five-degree digital fundus photographs (TRC-50LX; Topcon, Tokyo, Japan) and a semi-automated vessel measurement system (Retinal Analysis-IVAN, University of Wisconsin, Madison, Wisconsin, USA) were used to measure retinal vessel widths from imported fundus photographs^3^,^28^. The largest temporal arterial vessels (upper or lower) were selected in zone B. The arteries for IVAN analysis were the same as those chosen for AOSLO measurements.

### Reproducibility of AOSLO vascular measurements

ICCs were used to evaluate measurement reproducibility of the arteries selected for analyses. To evaluate inter-evaluator reproducibility of vascular calibers (OD, ID, WT, and WLR), the arteries of 20 normal subjects, at the same locations on the AOSLO images, were examined independently by two retina specialists in a masked manner. To evaluate inter-visit reproducibility, the same arteries were examined independently using two AOSLO images from 20 normal subjects collected on different days.

### Statistical analyses

All values are presented as mean ± standard deviation. Bivariate correlations were analyzed using the Pearson correlation coefficient. Paired *t*-tests were used to analyze vascular measurements, age, blood pressure parameters, and BMI. Unpaired *t*-tests were used to examine differences between measurements in normal and hypertensive participants aged >50 years. Multivariate regression analysis (backward stepwise method) was performed to identify predictors of WLR. All analyses were performed using StatView (Version 5.0; SAS Institute, Cary, NC), except for the ICC and multivariate regression, which were calculated using SPSS (v. 19; IBM Inc., Armonk, NY). Significance was set at *P *< 0.05.

## Additional Information

**How to cite this article**: Arichika, S. *et al.* Effects of age and blood pressure on the retinal arterial wall, analyzed using adaptive optics scanning laser ophthalmoscopy. *Sci. Rep.* **5**, 12283; doi: 10.1038/srep12283 (2015).

## Supplementary Material

Supplementary Information

## Figures and Tables

**Table 1:** Characteristics of the normal and hypertensive groups.

	**normal group**	**hypertensive group**	
No. patients	51	22	
20–29 years old	10	0	
30–39 years old	11	0	
40–49 years old	9	2	
50–59 years old	8	8	
60–69 years old	12	12	
70 years old	1	0	
Sex (male/female)	26/25	16/6	
Age (years)	44.3 ± 16.0	59.4 ± 6.7	p < 0.0001
Blood pressure (mmHg)			
Systolic	114.5 ± 10.9	140.0 ± 16.9	p < 0.0001
Diastolic	69.5 ± 9.2	84.1 ± 11.3	p < 0.0001
Pulse pressure (mmHg)	45.0 ± 6.3	55.9 ± 13.2	p < 0.0001
Heart rate (/min)	70.2 ± 11.3	70.1 ± 9.3	p = 0.85

**Table 2:** Characteristics of subjects aged >50 years in the normal and hypertensive groups.

	**Normal group**	**Hypertensive group**	***P* value**
Age (years)	60.6 ± 6.3	60.9 ± 4.7	0.87
Sex (male/female)	21(10/11)	20 (14/6)	
Outer diameter (μm)	128.2 ± 13.1	121.7 ± 19.2	0.21
Inner diameter (μm)	100.4 ± 11.5	92.9 ± 15.9	0.09
Wall thickness (μm)	27.8 ± 3.7	28.8 ± 5.6	0.50
Wall-to-lumen ratio	0.280 ± 0.040	0.315 ± 0.066	0.04
Body mass index	21.3 ± 2.2	23.0 ± 2.5	0.03
Axial length (mm)	24.2 ± 1.1	24.1 ± 1.0	0.77
Blood pressure (mmHg)			
Systolic	117.8 ± 11.3	139.9 ± 17.8	< 0.0001
Diastolic	71.7 ± 9.4	82.9 ± 11.3	<0.01
Pulse pressure (mmHg)	46.0 ± 5.7	56.9 ± 13.3	<0.01
Heart rate (/min)	71.6 ± 11.2	69.9 ± 9.7	0.62

Data are presented as the mean ± standard deviation.

**Table 3:** Correlation between vascular caliber and blood pressure parameters.

	**Outer diameter**		**Inner diameter**		**Wall thickness**		**Wall-to-lumen ratio**	
	**r**	***P***	**r**	***P***	**r**	***P***	**r**	***P***
Systolic blood pressure	−0.12	0.32	−0.33	<0.01	0.50	<0.0001	0.60	<0.0001
Diastolic blood pressure	−0.15	0.22	−0.37	0.001	0.54	<0.0001	0.65	<0.0001
Pulse pressure	−0.03	0.80	−0.13	0.29	0.24	0.04	0.27	0.02
Heart rate	0.20	0.09	0.15	0.21	0.18	0.12	0.05	0.65
Age	0.001	0.99	−0.21	0.07	0.54	<0.0001	0.56	<0.001
Body mass index	−0.05	0.66	−0.17	0.17	0.26	0.03	0.35	<0.01

**Table 4:** Multivariate correlations between wall-to-lumen ratio and independent variables.

**Parameter**	**Β**	**R**	**R^2^**	**Adjusted R^2^**	**P**
Model 1		0.645	0.416	0.407	
Diastolic blood pressure	0.645				<0.001
Model 2		0.727	0.529	0.514	
Diastolic blood pressure	0.490				<0.001
Age	0.370				<0.001

## References

[b1] Keith N. M., Wagener H. P. & Barker N. W. Some different types of essential hypertension: their course and prognosis. Am J Med Sci 268, 336–45 (1974). 4616627 10.1097/00000441-197412000-00004

[b2] Scheie H. G. Evaluation of ophthalmoscopic changes of hypertension and arteriolar sclerosis. AMA Arch Ophthalmol 49, 117–38 (1953). 13007237 10.1001/archopht.1953.00920020122001

[b3] Wong T. Y. *et al.* Computer-assisted measurement of retinal vessel diameters in the Beaver Dam Eye Study: methodology, correlation between eyes, and effect of refractive errors. Ophthalmology 111, 1183–90 (2004). 15177969 10.1016/j.ophtha.2003.09.039

[b4] Cheung C. Y., Ikram M. K., Sabanayagam C. & Wong T. Y. Retinal microvasculature as a model to study the manifestations of hypertension. Hypertension 60, 1094–103 (2012). 23045470 10.1161/HYPERTENSIONAHA.111.189142

[b5] Knudtson M. D. *et al.* Revised formulas for summarizing retinal vessel diameters. Curr Eye Res 27, 143–9 (2003). 14562179 10.1076/ceyr.27.3.143.16049

[b6] Schmieder R. E. & Ritt M. Wall-to-lumen ratio of retinal arterioles: a reproducible, valid and noninvasive approach for evaluation of early arteriolar changes in arterial hypertension *in vivo*. J Hypertens 30, 1108–10 (2012). 22573078 10.1097/HJH.0b013e328353f85a

[b7] Rizzoni D. *et al.* Relationship between media-to-lumen ratio of subcutaneous small arteries and wall-to-lumen ratio of retinal arterioles evaluated noninvasively by scanning laser Doppler flowmetry. J Hypertens 30, 1169–75 (2012). 22504847 10.1097/HJH.0b013e328352f81d

[b8] Harazny J. M. *et al.* Increased wall:lumen ratio of retinal arterioles in male patients with a history of a cerebrovascular event. Hypertension 50, 623–9 (2007). 17698722 10.1161/HYPERTENSIONAHA.107.090779

[b9] Lehmann M. V. & Schmieder R. E. Remodeling of retinal small arteries in hypertension. Am J Hypertens 24, 1267–73 (2011). 21956527 10.1038/ajh.2011.166

[b10] Ritt M. & Schmieder R. E. Wall-to-lumen ratio of retinal arterioles as a tool to assess vascular changes. Hypertension 54, 384–7 (2009). 19451413 10.1161/HYPERTENSIONAHA.109.133025

[b11] Muraoka Y. *et al.* Age- and hypertension-dependent changes in retinal vessel diameter and wall thickness: an optical coherence tomography study. Am J Ophthalmol 156, 706–14 (2013). 23876868 10.1016/j.ajo.2013.05.021

[b12] Chui T. Y., Vannasdale D. A. & Burns S. A. The use of forward scatter to improve retinal vascular imaging with an adaptive optics scanning laser ophthalmoscope. Biomed Opt Express 3, 2537–49 (2012). 23082294 10.1364/BOE.3.002537 PMC3470005

[b13] Chui T. T. & Lee W. C. A regression-based method for estimating risks and relative risks in case-base studies. PLoS One 8, e83275 (2013). 24349478 10.1371/journal.pone.0083275 PMC3861498

[b14] Koch E. *et al.* Morphometric analysis of small arteries in the human retina using adaptive optics imaging: relationship with blood pressure and focal vascular changes. J Hypertens 32, 890–8 (2014). 24406779 10.1097/HJH.0000000000000095 PMC3966915

[b15] Roorda A. & Williams D. R. The arrangement of the three cone classes in the living human eye. Nature 397, 520–2 (1999). 10028967 10.1038/17383

[b16] Dubra A. *et al.* Noninvasive imaging of the human rod photoreceptor mosaic using a confocal adaptive optics scanning ophthalmoscope. Biomed Opt Express 2, 1864–76 (2011). 21750765 10.1364/BOE.2.001864 PMC3130574

[b17] Takayama K. *et al.* High-resolution imaging of the retinal nerve fiber layer in normal eyes using adaptive optics scanning laser ophthalmoscopy. PLoS One 7, e33158 (2012). 22427978 10.1371/journal.pone.0033158 PMC3299751

[b18] Martin J. A. & Roorda A. Pulsatility of parafoveal capillary leukocytes. Exp Eye Res 88, 356–60 (2009). 18708051 10.1016/j.exer.2008.07.008 PMC2696158

[b19] Tam J. *et al.* Disruption of the retinal parafoveal capillary network in type 2 diabetes before the onset of diabetic retinopathy. Invest Ophthalmol Vis Sci 52, 9257–66 (2011). 22039250 10.1167/iovs.11-8481 PMC3302433

[b20] Arichika S., Uji A., Ooto S., Miyamoto K. & Yoshimura N. Adaptive optics-assisted identification of preferential erythrocyte aggregate pathways in the human retinal microvasculature. PLoS One 9, e89679 (2014). 24586959 10.1371/journal.pone.0089679 PMC3935927

[b21] Bedggood P. & Metha A. Direct visualization and characterization of erythrocyte flow in human retinal capillaries. Biomed Opt Express 3, 3264–77 (2012). 23243576 10.1364/BOE.3.003264 PMC3521302

[b22] Schiffrin E. L. Vascular remodeling in hypertension: mechanisms and treatment. Hypertension 59, 367–74 (2012). 22203749 10.1161/HYPERTENSIONAHA.111.187021

[b23] Baleanu D. *et al.* Wall-to-lumen ratio of retinal arterioles and arteriole-to-venule ratio of retinal vessels in patients with cerebrovascular damage. Invest Ophthalmol Vis Sci 50, 4351–9 (2009). 19339746 10.1167/iovs.08-3266

[b24] Cuspidi C. & Sala C. Retinal wall-to-lumen ratio: a new marker of endothelial function? J Hypertens 29, 33–5 (2011). 21160362 10.1097/HJH.0b013e328341c6b4

[b25] Ritt M. *et al.* Analysis of retinal arteriolar structure in never-treated patients with essential hypertension. J Hypertens 26, 1427–34 (2008). 18551020 10.1097/HJH.0b013e3282ffdc66

[b26] Wong T. Y., Klein R., Klein B. E., Meuer S. M. & Hubbard L. D. Retinal vessel diameters and their associations with age and blood pressure. Invest Ophthalmol Vis Sci 44, 4644–50 (2003). 14578380 10.1167/iovs.03-0079

[b27] Michelson G. *et al.* Morphometric age-related evaluation of small retinal vessels by scanning laser Doppler flowmetry: determination of a vessel wall index. Retina 27, 490–8 (2007). 17420704 10.1097/01.iae.0000243032.33738.f7

[b28] Hubbard L. D. *et al.* Methods for evaluation of retinal microvascular abnormalities associated with hypertension/sclerosis in the Atherosclerosis Risk in Communities Study. Ophthalmology 106, 2269–80 (1999). 10599656 10.1016/s0161-6420(99)90525-0

[b29] Edwards B. S., Lucas R. V. Jr., Lock J. E. & Edwards J. E. Morphologic changes in the pulmonary arteries after percutaneous balloon angioplasty for pulmonary arterial stenosis. Circulation 71, 195–201 (1985). 3155497 10.1161/01.cir.71.2.195

[b30] Bennett A. G., Rudnicka A. R. & Edgar D. F. Improvements on Littmann’s method of determining the size of retinal features by fundus photography. Graefes Arch Clin Exp Ophthalmol 232, 361–7 (1994). 8082844 10.1007/BF00175988

[b31] Arichika S. *et al.* Retinal hemorheologic characterization of early-stage diabetic retinopathy using adaptive optics scanning laser ophthalmoscopy. Invest Ophthalmol Vis Sci 55, 8513–22 (2014). 25212778 10.1167/iovs.14-15121

[b32] Mancia G. *et al.* 2013 ESH/ESC Guidelines for the management of arterial hypertension: the Task Force for the management of arterial hypertension of the European Society of Hypertension (ESH) and of the European Society of Cardiology (ESC). J Hypertens 31, 1281–357 (2013). 23817082 10.1097/01.hjh.0000431740.32696.cc

[b33] M O.H., O’Donoghue E. & Dainty C. Measurement of the retinal arteriolar response to a hyperoxic provocation in nonsmokers and smokers, using a high-resolution confocal scanning laser ophthalmoscope. J Biomed Opt 19, 076012 (2014). 25023416 10.1117/1.JBO.19.7.076012

[b34] Chapman N. *et al.* Computer algorithms for the automated measurement of retinal arteriolar diameters. Br J Ophthalmol 85, 74–9 (2001). 11133716 10.1136/bjo.85.1.74 PMC1723694

