# Supplementary material for: Effects of age and blood pressure on the retinal arterial wall, analyzed using adaptive optics scanning laser ophthalmoscopy
Source: Sci Rep. 2015 Jul 20;5:12283. doi: 10.1038/srep12283 (PMC4507481; doi:10.1038/srep12283)

Effects of age and blood pressure on the retinal arterial wall, analyzed using adaptive optics scanning laser ophthalmoscopy

Shigeta Arichika, Akihito Uji, Sotaro Ooto, Yuki Muraoka, and Nagahisa Yoshimura

The Department of Ophthalmology and Visual Sciences, Kyoto University Graduate School of Medicine, Kyoto 606-8507, Japan

Figure legends.

Supplementary Figure S1. Vascular wall border detection and wall thickness measurement. (a) The seed points were manually set at the center of the artery along its running direction (blue line). (b) A sliding linear regression filter (SLRF) with a larger window size (W = 10 pixels) was used to determine the approximate position of the vascular region. (c) The outliers were then deleted. (d) After using the natural spline interpolation method, interpolated candidate points indicated the approximate position of the vascular wall. (e) A SLRF with a smaller window size (window size [W] = 4 pixels) was used in order to determine the precise position of the vascular region. (f) The outlying vascular wall border candidate points were deleted. (g) The natural spline interpolation method was used to determine the remaining vascular wall border candidates. (h) The automated wall border detection was completed. Green and pink lines indicate the outer wall border, and yellow and orange lines indicate the inner wall border. Continuous measurements were performed automatically at 6 µm intervals along the segmented border lines.

Supplementary Figure S2. Vascular wall border detection step with a sliding linear regression filter (SLRF). (a) AOSLO image with clear arterial wall visualization. (b) Intensity profile for the green line on panel (a). Yellow zones represent the arterial wall location. (c) Intensity profile filtered by SLRF (window size [W] = 10 pixels). The approximate position of the vascular wall region can be determined by finding the minimum point in the left side and the maximum point in the right side from the zero-cross point corresponding to the central axis. (d) Intensity profile filtered by a SLRF (W = 4 pixels). We select the 2 nearest extremal points to each first candidate point as vascular wall border candidate points.

Supplementary Figure S1.

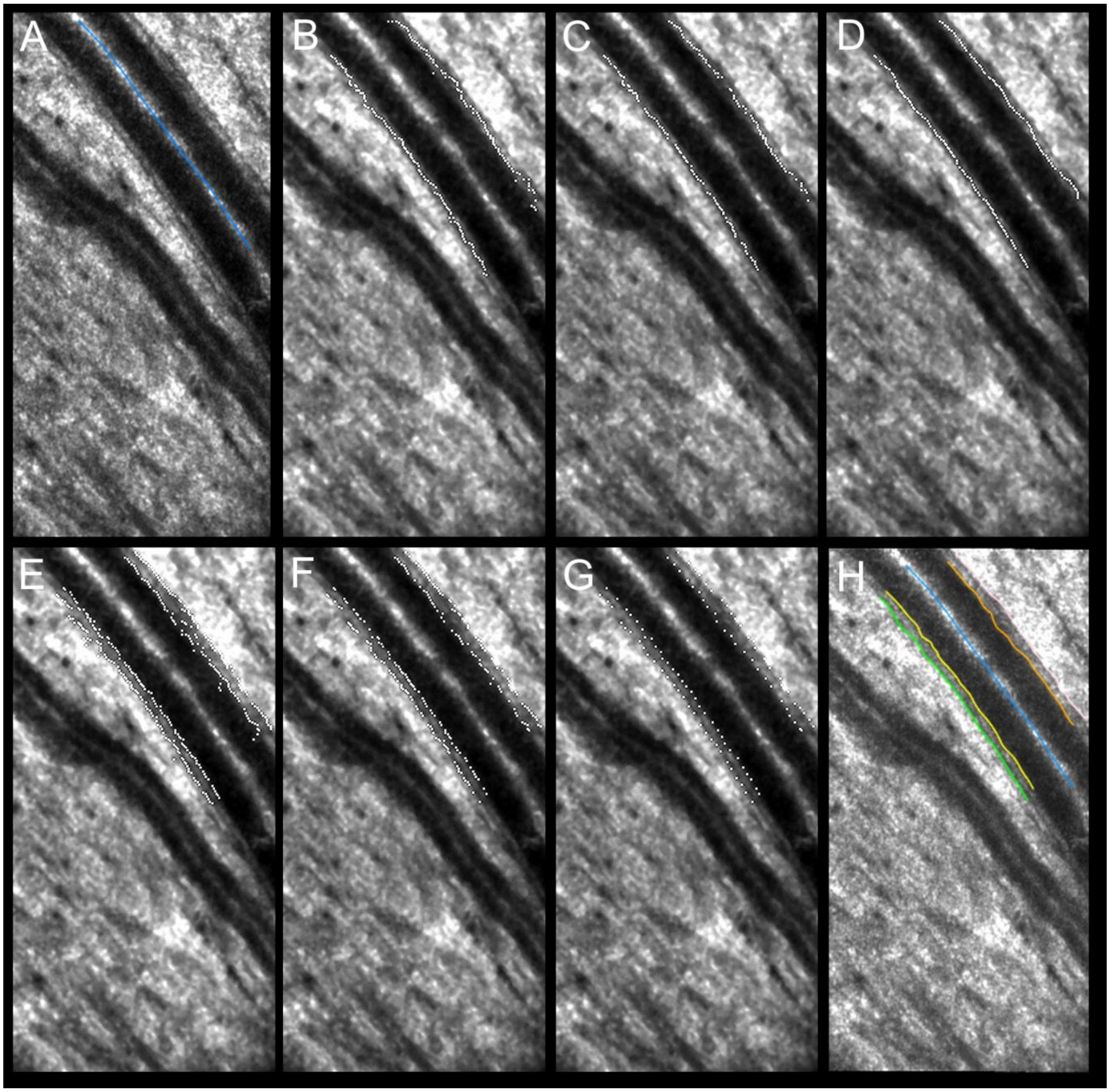

Supplementary Figure S2.

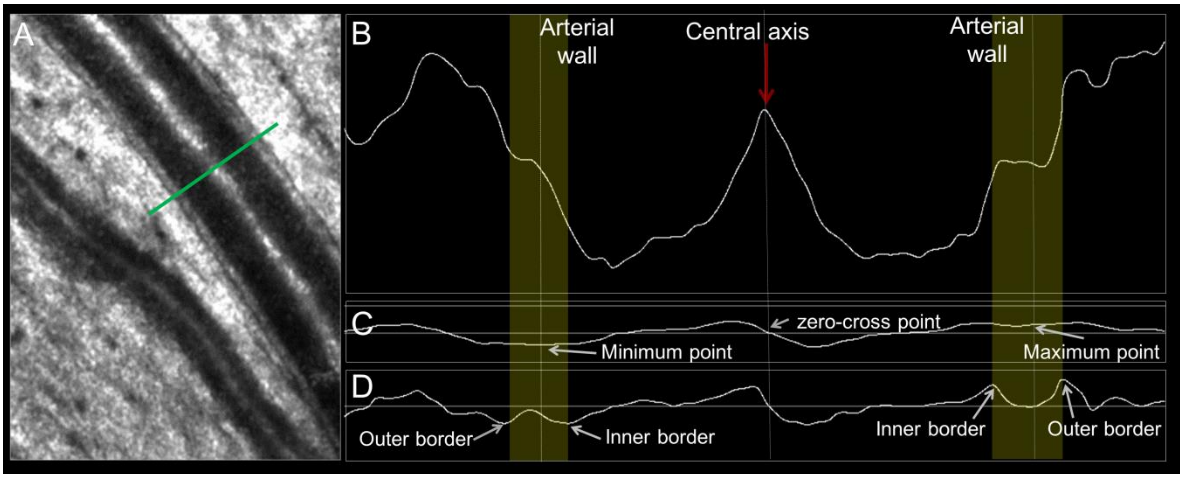

Supplement: Supplementary Information [file srep12283-s1.pdf]
